# Genetic Algorithm-Based Cooperative Coding and Caching Data Dissemination Scheme in Multi-UAV-Enabled Internet of Vehicles

**DOI:** 10.3390/s24144443

**Published:** 2024-07-09

**Authors:** Ke Xiao, Jie Hu, Chunlin Li, Wenjie Ji, Jinkun Xu, Huang Du

**Affiliations:** 1 Department of Computer and Information Science, Chongqing Normal University, Chongqing 401331, China; 2023210516037@stu.cqnu.edu.cn (J.H.); 2023110516059@stu.cqnu.edu.cn (C.L.); 2023210516039@stu.cqnu.edu.cn (W.J.); 2023110516069@stu.cqnu.edu.cn (J.X.); 2 Chongqing Planning Exhibition Gallery (Chongqing Planning Research Institute), Chongqing 400060, China; ghyjzx@126.com

**Keywords:** data dissemination, unmanned aerial vehicle, network coding, Internet of Vehicles

## Abstract

Unmanned Aerial Vehicles (UAVs) have emerged as efficient tools in disaster-stricken areas, facilitating efficient data dissemination for post-disaster rescue operations. However, the limited onboard energy of UAVs imposes significant constraints on their operational lifespan, thereby presenting substantial challenges for efficient data dissemination. Therefore, this work investigates a data dissemination scheme to enhance the UAVs’ bandwidth efficiency in multi-UAV-enabled Internet of Vehicles, thereby reducing UAVs’ energy consumption and improving overall system performance when UAVs hover along designated flight trajectories for data dissemination. Specifically, first, we present a software-defined network-based framework for data dissemination in multi-UAV-enabled IoV. According to this framework, we formulate a problem called C2BS (Coding-based Cooperative Broadcast Scheduling) that focuses on optimizing the UAVs’ bandwidth efficiency by leveraging the combined benefits of coding and caching. Furthermore, we demonstrate the NP-hardness of the C2BS problem by employing a polynomial time reduction technique on the simultaneous matrix completion problem. Then, inspired by the benefits offered by genetic algorithms, we propose a novel approach called the Genetic algorithm-based Cooperative Scheduling (GCS) algorithm to address the C2BS problem. This approach encompasses a coding scheme for representing individuals, a fitness function for assessing individuals, operators (i.e., crossover and mutation) for generating offspring, a local search technique to enhance search performance, and a repair operator employed to rectify infeasible solutions. Additionally, we present an analysis of the time complexity for the GCS algorithm. Finally, we present a simulation model to evaluate the performance. Experimental findings provide evidence of the excellence of the proposed scheme.

## 1. Introduction

With the rapid advancement of communication technologies and the proliferation of mobile applications, Internet of Vehicles (IoV) [1,2] has emerged as a promising concept for achieving Intelligent Transportation Systems (ITSs), encompassing advancements in improving safety and security [3], enhancing driving experience [4], and providing entertainment services [5]. Data dissemination through Vehicle-to-Vehicle (V2V) and Infrastructure-to-Vehicle (I2V) communications plays a pivotal role in diverse ITSs through Vehicle-to-Vehicle (V2V) and Infrastructure-to-Vehicle (I2V) communications. However, the performance of data dissemination is expected to decline because of the highly dynamic characteristics of vehicles [1] in IoV, particularly in complex environments resulting from natural disasters such as wildfires, storms, and earthquakes. On the one hand, infrastructures for wireless communication, including Base Stations (BSs) and RoadSide Units (RSUs), will be unable to perform normally to provide data service via I2V communication, since they may be destroyed in natural disasters. Poor network connectivity in highly dynamic communication environments may hinder data dissemination through V2V communication, resulting in intermittent communication connections and high end-to-end latency. On the other hand, for natural disasters, effective post-disaster rescue heavily relies on fast response to the disaster with low-latency data dissemination [6]. Hence, data dissemination in disaster scenarios, where the existing infrastructure is unavailable or incapacitated, poses significant challenges that hinder effective support for post-disaster rescue efforts.

Unmanned Aerial Vehicles (UAVs) have been employed to enhance communication connectivity among vehicles and establish UAV-enabled IoV to improve network performance [7,8]. The primary motivation behind this stems from their inherent characteristics, which include exceptional flexibility and cost-effectiveness in deployment [9]. Inspired by this concept, UAVs have the potential to serve as airborne base stations or relay nodes for transmitting rescue messages to vehicles in areas impacted by natural disasters. This capability empowers UAVs to disseminate scheduling commands for post-disaster rescue operations to ground vehicles. However, the limited onboard energy of UAVs necessitates special consideration regarding the energy issue in UAV-enabled IoV. The energy consumption of UAVs primarily comprises propulsion (i.e., hovering and flying) energy and communication energy [10]. The bandwidth efficiency of UAVs for data dissemination becomes crucial in influencing the overall energy consumption of UAVs when they hover along designated flight trajectories for a specific duration. For instance, if UAVs broadcast with constant power consumption, improving the bandwidth efficiency of UAVs can effectively reduce the number of broadcasting and service latency, thereby mitigating energy consumption aspects such as hovering and communication. Therefore, optimizing the bandwidth efficiency of UAVs for data dissemination facilitates efficient utilization of energy resources, thereby enhancing the endurance capabilities of UAVs and the overall system performance. Although the detailed methods for UAV deployment are beyond the scope of this research, they can be referenced from previous studies [7,11]. As a result, this work aims to develop an effective data dissemination strategy that optimizes bandwidth efficiency for UAVs, enhancing UAV endurance capabilities and overall system performance in post-disaster rescue operations.

Relevant efforts have been dedicated to enhancing the system performance of wireless communication. On the one hand, the potential of network coding can greatly improve data dissemination performance, such as data rate and bandwidth efficiency [12,13,14]. Due to sufficient storage of vehicles to cache data items, it is possible to improve the gain of network coding by coordinating the strategy of coding and caching. Compared to a system with only one UAV, where service capacity is constrained, multi-UAV cooperative service can significantly improve network performance. To achieve this, this work focuses on investigating and developing a coding-based scheduling algorithm for UAVs, aiming to optimize bandwidth efficiency by harnessing the combined advantages of coding and caching. On the other hand, great interest has been aroused in research on the service architecture by integrating Software-Defined Network (SDN) into IoV since SDN has shown capacities of flexible resource allocation and service management [15,16]. The SDN framework aims at separating the network into control and data planes. Leveraging the global view of the entire network, the SDN controller facilitates flexible management for making scheduling decisions to improve network performance through its logically centralized control capabilities.

With the above motivation, this study proposes an SDN-based data dissemination architecture in multi-UAVs-enabled IoV to facilitate scheduling decisions for UAVs in a logically centralized manner, which lays the foundation for leveraging coding and caching to provide efficient data service in IoV. Moreover, inspired by the advantages of genetic algorithms for dealing with complicated optimization issues, this study presents a genetic algorithm-based scheduling scheme for data dissemination implemented at the SDN controller, aiming to enhance UAVs’ bandwidth efficiency and optimize the overall performance of data dissemination. The primary contributions of this work are outlined as follows.

- We propose an SDN-based data dissemination framework in multi-UAV-enabled IoV. Specifically, the SDN controller leverages the knowledge from the entire network, encompassing motion information, requested information, and caching information of vehicles, to generate coding-based scheduling strategies for UAVs. Then, UAVs execute these scheduling decisions to facilitate efficient data dissemination for vehicles.
- Based on this framework, a novel problem called Coding-based Cooperative Broadcast Scheduling (C2BS) is formulated to optimize the UAVs’ bandwidth efficiency by considering the combined advantages of coding and caching at the SDN controller.
- The Np-hardness of the C2BS problem is proven by employing a polynomial time reduction technique on the problem of simultaneous matrix completion, which is known to be an NP-complete problem over GF(2) [17].
- Inspired by the benefits offered by genetic algorithms, we propose a Genetic algorithm-based Cooperative Scheduling (GCS) algorithm that utilizes genetic algorithms to tackle the C2BS problem. This algorithm incorporates various operators, including solution representation, solution evaluation, solution evolution, and adjustment of infeasible solutions.
- We develop a simulation model employing the proposed system framework, and perform an extensive performance evaluation by implementing the GPS data of taxis. Experimental findings show the effectiveness of the proposed scheme.

The remaining sections of this paper are organized as follows. The literature review is conducted in Section 2. We propose the system model in Section 3. Section 4 formulates the C2BS problem and establishes the NP-hardness of this problem. The GCS algorithm is proposed in Section 5, where we also analyze its time complexity. In Section 6, we perform the performance evaluation to validate the proposed scheme. we provide the conclusion in Section 7.

## 2. Related Work

Extensive studies have been conducted to explore the implementation of UAVs to enhance network performance. Zhan et al. [18] examined a communication system involving a UAV serving as a mobile BS to receive data from ground facilities. They proposed an optimization algorithm that utilizes path discretization and successive convex approximation to optimize the throughput of data uploading. An architecture aiming to optimize vehicular computation offloading using SDN and UAV assistance was proposed by Zhao et al. [19], wherein the system cost is minimized through deploying a UAV as a relay node. Yang et al. [20] investigated a system that combines UAVs with mobile edge computing, enabling a single UAV to cater to several energy-harvesting devices. To optimize energy consumption and task processing rate, they developed an innovative online algorithm that incorporates perturbed Lyapunov optimization. Dai et al. [21] studied a framework for offloading vehicular tasks with the assistance of UAVs, aiming to tackle the overloaded issue in vehicular edge computing. To reduce delays in vehicular tasks, they introduced an online algorithm for task offloading that utilizes Markov approximation optimization while considering energy limitations imposed on the UAV. Yan et al. [22] introduced a UAV-assisted mobile edge computing system for the IoV, and proposed a task offloading algorithm that integrates deep reinforcement learning into vehicular edge computing to minimize delays in the system. The above works consider scenarios involving only one single UAV. However, for complicated post-disaster rescue operations, the requirements of large-scale servers cannot be fulfilled solely by relying on a single UAV. Therefore, this work aims to enhance data dissemination performance in challenging scenarios by leveraging the capabilities of multiple UAVs.

Considerable efforts have been devoted to investigating the utilization of multiple UAVs in UAV-enabled IoV. Sami et al. [23] proposed a framework that employs UAVs for vehicular fog computing, aiming to enhance cluster management efficiency and service restoration, thereby improving the quality of service in the automotive domain. Ning et al. [24] studied the management of distributed multi-UAV trajectory control within UAV-assisted mobile edge computing networks, aiming to optimize users’ short-term computational cost and minimize UAVs’ long-term computational cost while delivering differentiated services. Liu et al. [25] proposed a mobile IoV framework that utilizes multiple UAVs to offer tracking services for vehicles. These UAVs transmit downlink information during flight to optimize system throughput by jointly optimizing vehicle communication scheduling, UAV power allocation, and UAV trajectory. Due to the absence of collaborative service-oriented modeling in distributed network settings, Kumar et al. [26] introduces a framework for ensuring quality of service in a UAV-supported aerial ad hoc network environment to devote to reliable aerial communication. In the context of emergency communication with the assistance of UAVs, Wang et al. [27] presented an authentication and key agreement protocol, which utilizes three components, namely smart cards, biometrics, and passwords along with the physically unclonable function to ensure the security of UAV-assisted emergency communication. Nevertheless, in UAV-enabled IoV, the energy constraints of UAVs pose significant challenges to the performance of data dissemination. Enhancing the UAVs’ bandwidth efficiency becomes imperative for improving their endurance capabilities. Consequently, this work focuses on devising a data dissemination scheme for multiple UAVs to maximize bandwidth efficiency and optimize overall energy consumption.

Many studies have focused on exploiting network coding to enhance the overall performance of IoV. To enhance spectrum efficiency in IoV, Pazhoor et al. [12] proposed an index-coded Non-Orthogonal Multiple Access (NOMA) technique, which effectively minimizes the transmission count by integrating NOMA techniques with index-coded data. The practical implementation of DNN-PNC, which is based on Physical-layer Network Coding (PNC) and utilizes Deep Neural Network (DNN), was investigated by Wang et al. [13]. They used the universal software radio peripheral platform to demonstrate the feasibility of employing DNN-based PNC for IoV. The frequent transmission errors lead to underutilization of channel resources, Pan et al. [14] studied a Ggraph-Theoretic method-based Network Coding (GTNC) approach to enhance resilience against transmission errors and proposed a GTNC-based cooperative concurrent distribution solution to coordinate V2V and I2V communications. To leverage the benefits of network coding, Liu et al. [28] explored the integration of fog computing into a software-defined heterogeneous IoV architecture and proposed a scheduling algorithm that utilizes clique-searching to tackle the cooperative service problem in fog-assisted environments, to minimize service delay. Bhatia et al. [29] presented a server architecture that utilizes network coding and incorporates multi-generation-mixing capabilities to improve the reliability and security of data transmission. Huang et al. [30] investigated the efficient allocation of system resources in an IoV system with multiple users by utilizing network coding. However, the performance of the coding-based scheme is significantly influenced by the crucial incorporation of caching. Therefore, this study aims to investigate a scheduling strategy that integrates coding and caching to enhance the data dissemination performance of UAVs in UAV-enabled IoV.

Distinguishing from prior research, this work focuses on exploiting multiple UAVs for data dissemination in post-disaster rescue operations. Considering UAVs have limited energy capacities and hover along designated flight trajectories for data dissemination, we are committed to maximizing the bandwidth efficiency of UAVs to enhance their endurance capabilities. Consequently, we propose an SDN-based data dissemination architecture in multi-UAV-enabled IoV and develop a customized scheduling algorithm for UAVs that leverages the combined benefits of coding and caching aiming to optimize the bandwidth resources of UAVs, thereby enhancing the overall performance of the system.

## 3. System Model

**Figure 1:**
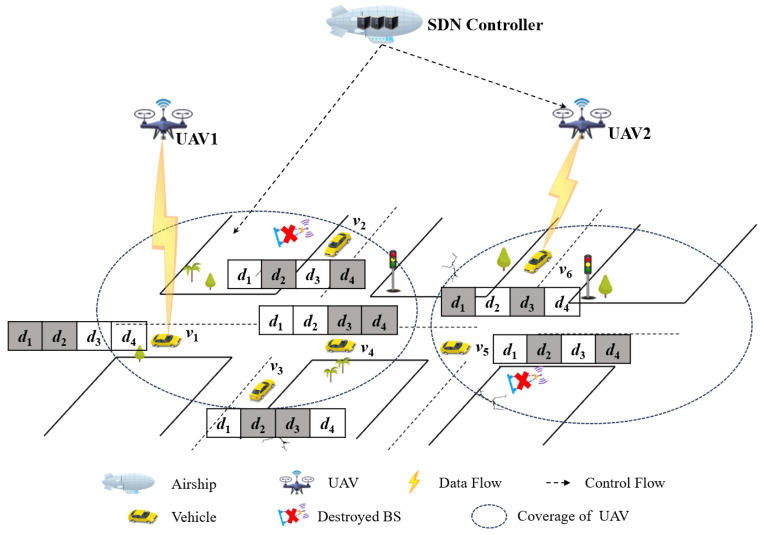
SDN-based data dissemination framework in multi-UAV-enabled IoV.

The SDN-based data dissemination framework in multi-UAV-enabled IoV is illustrated in Figure 1. In this framework, vehicles, and UAVs are regarded as switches in the data plane. The airship, equipped with a cellular network interface, serves as the SDN controller responsible for establishing the control plane to gather vehicle and UAV status data and oversee the entire network. Then, the SDN controller leverages the global view of the entire network to formulate and implement scheduling decisions encompassing resource allocation, routing determinations, and data scheduling. Data nodes perform scheduling decisions forwarded from the control plane.

We consider a disaster-stricken area, where infrastructure is unavailable. Vehicles request data services for collective interests in post-disaster rescue operations, such as disaster information, road conditions, and weather updates. UAVs facilitate the provision of this information to vehicles through wireless communication. Note that a portion of the requested data might have been cached by vehicles before they entered the scenario. Based on this, UAVs store the requested data and are deployed and maintained in a hovering state for a specific duration to facilitate data dissemination for vehicles. It should be noted that the deployment methods of UAVs can be referenced from the literature mentioned in Section 1. We are dedicated to investigating a scheduling strategy that maximizes UAVs’ bandwidth efficiency during the hovering state for data dissemination to enhance overall system performance. Subsequent redeployment of UAVs can follow predetermined trajectories. Additionally, vehicles in the coverage of UAV (indicated by the dashed circular shape shown in Figure 1) can obtain data via the equipped cellular network interfaces. To this goal, we propose a framework for UAV data dissemination that utilizes SDN to exploit centralized control and flexible scheduling.

The operational flow of the system is delineated as follows. First, vehicles and UAVs periodically transmit their status information, including location, velocity, direction, requested contents, and cached contents to the SDN controller via control messages upon entering the designated area. Furthermore, the SDN controller with a perspective of the entire network can predict vehicle trajectories, make informed scheduling decisions, and transmit control messages to UAVs. Consequently, UAVs possess the capacity to make scheduling decisions and provide data dissemination services for vehicles. A scheduling strategy is designed and implemented in the SDN controller, which utilizes network coding to efficiently facilitate data dissemination for vehicles. Note that detailed prediction solutions are beyond the scope of this study, they can be referenced from previous works [31,32].

The above framework serves as the fundamental basis for implementing network coding through informed scheduling decisions to enhance the performance of data dissemination. Given this, the SDN controller utilizes a technology based on bitwise exclusive-or (⊕) coding. The reason is as follows. First, the utilization of XOR coding-based operators incurs negligible overhead, thereby facilitating handling by UAVs. Moreover, the size of the encoded packet remains unchanged compared to that of the original one. For instance, given a packet *p* = $d_{1}⊕d_{2}$, where data items $d_{1}$ and $d_{2}$ are used for encoding, a vehicle cached one data item (e.g., $d_{1}$) can decode the other data item (e.g., $d_{2}$) in packet *p* by performing the computation of $d_{2}=d_{1}⊕p$. By scheduling an encoded packet, it becomes feasible to cater to multiple requested data items from diverse vehicles. Additionally, in comparison to traditional wireless sensor networks, vehicles have a higher capability for data caching and processing. Consequently, the encoded packets that cannot be immediately decoded can be cached by these vehicles.

We illustrate the proposed framework and challenges of scheduling algorithm design through an exemplification. As shown in Figure 1, suppose the database consists of $d_{1}$, $d_{2}$, $d_{3}$, and $d_{4}$, two UAVs, namely UAV_1_ and UAV_2_, are deployed to facilitate data dissemination for a fleet of six vehicles (denoted as $v_{1}$, $v_{2}$, $v_{3}$, $v_{4}$, $v_{5}$, and $v_{6}$). Furthermore, assuming a packet transmission time of 1-time slot and a maximum scheduling period of 3-time slots, it can be inferred that each UAV can broadcast up to three packets within every scheduling period. In addition, the gray square shows the cached data item. As shown in Figure 1, at time $t_{0}$, the current cached packets are $d_{1}$ and $d_{2}$ for $v_{1}$, $d_{2}$ and $d_{4}$ for $v_{2}$, $d_{2}$ and $d_{3}$ for $v_{3}$, $d_{3}$ and $d_{4}$ for $v_{4}$, $d_{2}$ and $d_{4}$ for $v_{5}$, and $d_{1}$ and $d_{3}$ for $v_{6}$. Meanwhile, $v_{1}$, $v_{2}$, $v_{3}$, $v_{4}$ are in the coverage of UAV_1_, $v_{5}$, $v_{6}$ are in the coverage of UAV_2_. Table 1 presents the results of vehicle trajectory prediction.

Given the above settings, we analyze the performance of three scheduling strategies on average service delay. The average delay is defined by the total time elapsed by all vehicles to obtain all data items in the database, divided by the total count of vehicles. Table 2 presents scheduling strategies and results. The detailed explanation of the scheduling process is elaborated upon in the following manner.

- Independent Scheduling (IS): In this scenario, UAVs independently provide data services without considering network coding. As illustrated in Table 2, UAV_1_ broadcasts $d_{1}$, $d_{3}$ at times $t_{0}$, $t_{1}$, respectively, while UAV_2_ broadcasts $d_{1}$, $d_{2}$ and $d_{4}$ at times $t_{0}$, $t_{1}$ and $t_{2}$, respectively. Taking $v_{5}$ as an exemplification, it receives $d_{1}$ broadcast by UAV_2_ at $t_{0}$. UAV_2_ broadcasts $d_{2}$ and $d_{4}$ at time $t_{1}$ and $t_{2}$, respectively. Except for vehicle $v_{5}$, all other vehicles can be completely served, requiring an additional time slot to broadcast $d_{3}$ for serving $v_{5}$. The vehicles experience service delays of 3, 2, 3, 2, 4, and 3 time slots. As a result, the average delay for the IS is approximately 2.8 time slots.
- Coding-based Individual Scheduling (CIS): In this scenario, UAVs broadcast individually by implementing network coding. As shown in Table 2, UAV_1_ broadcasts $d_{1}⊕d_{4}$ and $d_{2}⊕d_{3}$ at times $t_{0}$ and $t_{1}$, respectively, while UAV_2_ broadcasts $d_{1}⊕d_{2}$, $d_{3}⊕d_{4}$ and $d_{1}⊕d_{2}$ at times $t_{0}$, $t_{1}$ and $t_{2}$, respectively. Taking $v_{3}$ as an exemplification, at time $t_{0}$, because the encoded packet $d_{1}⊕d_{4}$ broadcast by UAV_1_ cannot be decoded, $v_{3}$ cannot be served. Then, it can decode out $d_{4}$ from $d_{3}⊕d_{4}$ broadcast by UAV_2_ at time $t_{1}$. To completely serve $v_{3}$ and $v_{4}$, UAV_2_ has to broadcast $d_{1}⊕d_{2}$ at time $t_{2}$. To provide complete service to all vehicles, it is necessary to schedule 3 time slots, resulting in delays of 2, 2, 3, 3, 2, and 2 time slots. This leads to an average delay of approximately 2.3 time slots for the CIS.
- Coding-based Cooperative Scheduling (CCS): In this scenario, the scheduling of UAVs is optimized by leveraging the advantages of coding and caching. In scheduling process, UAV_1_ broadcasts $d_{1}⊕d_{4}$ for $v_{1}$, $v_{2}$, $v_{3}$, $v_{4}$ at time $t_{0}$ and $d_{3}$ for $v_{1}$, $v_{2}$ at time $t_{1}$, respectively. UAV_2_ broadcasts $d_{3}⊕d_{4}$ for $v_{5}$, $v_{6}$ at time $t_{0}$ and $d_{1}⊕d_{2}$ for $v_{3}$, $v_{4}$, $v_{5}$, $v_{6}$ at time $t_{1}$, respectively. The scheduling procedure is outlined in Table 2. Taking $v_{3}$ as an exemplification, $d_{1}⊕d_{4}$ broadcast by UAV_1_ cannot be immediately decoded at time $t_{0}$, but $d_{1}⊕d_{4}$ will be stored by $v_{3}$ since with the vehicle trajectory prediction, $v_{3}$ will enter into the coverage area of UAV_2_ at $t_{1}$, and the $d_{1}⊕d_{2}$ will be broadcast by UAV_2_. Then, $v_{3}$ can retrieve $d_{1}$ when $d_{1}⊕d_{2}$ is broadcast by UAV_2_. Meanwhile, $v_{3}$ can obtain $d_{4}$ by decoding previously cached $d_{1}⊕d_{4}$. All vehicles receive service within 2 time slots, with a delay of 2 time slots per vehicle. Consequently, the average delay for the CCS is merely 2 time slots.

## 4. Problem Analysis

### 4.1. Problem Formulation

Let $V=\{v_{1},v_{2},\ldots ,v_{\left|V\right|}\}$ represent the set of vehicles, where |V| denotes the overall count of vehicles. The set of UAVs is symbolized as $U=\{u_{1},u_{2},\ldots ,u_{\left|U\right|}\}$, where |U| denotes the total count of UAVs. Let $V_{j}=\left\{v_{1}^{j},v_{2}^{j},\ldots ,v_{|V_{j}|}^{j}\right\}$ ($V_{j}\subseteq V$) denote the set of vehicles that fall within the radio coverage range of UAV $u_{j}$ ($u_{j}\in U$). The set of data items is designed as $D=\{d_{1},d_{2},\ldots ,d_{\left|D\right|}\}$, where |D| represents the overall count of data items in the database. In the XOR-based coding scheme, the encoded packet *p* can be represented by a coefficient vector a(p) of dimension |D|, denoted by $a\left(p\right)=\{a{\left(p\right)}_{1},a{\left(p\right)}_{2},\ldots ,a{\left(p\right)}_{\left|D\right|}\}$, where each element $a{\left(p\right)}_{i}$ is a binary variable. For instance, the element $a{\left(p\right)}_{i}$ in a(p) will be assigned a value of 1 if the data item $d_{i}$ ($d_{i}\in D$) is included in the encoding process of *p*, and 0 otherwise. Similarly, a non-encoded data item $d_{i}$ ($d_{i}\in D$) can alternatively be transformed into a coefficient vector a(p) of dimension |D|, where only the corresponding element $a{\left(p\right)}_{i}$ is set to 1 while all other elements are set to 0. Hence, in this paper, the term ‘packet’ can be used interchangeably to refer to both encoded and non-encoded packets without causing any confusion. In addition, we define the cached packet set of a vehicle (say $v_{k}$ ($v_{k}\in V$) ) as $C\left(v_{k}\right)=\left\{p_{1}^{k},p_{2}^{k},\ldots ,p_{\left|C(v_{k})\right|}^{k}\right\}$, where $\left|C\left(v_{k}\right)\right|$ denotes the total count of packets cached by vehicle $v_{k}$. The essential symbols in this paper are outlined in Table 3.

The C2BS problem is formulated based on the aforementioned illustration. Considering the set of vehicles *V*, the set of data items *D*, the set of packets cached by each vehicle $C\left(v_{k}\right)\left(v_{k}\in V\right)$, and the set of UAVs *U*, the C2BS problem aims at maximizing UAVs’ bandwidth efficiency by identifying a minimal group of packets, denoted as *P*, that can cater to all vehicles.

### 4.2. NP-Hardness Proof

In this section, we prove that the C2BS problem is an NP-hard problem by employing a polynomial-time reduction technique, which is applied to the simultaneous matrix completion problem known to be an NP-complete problem over GF(2). First, we provide an overview of the basic idea behind the problem of simultaneous matrix completion. The matrices in question consist of a mix of numerical values and symbolic variables, where each symbolic variable is exclusive to one matrix but may appear in multiple matrices. Then, this problem aims to simultaneously attain complete rank for all matrices by determining and assigning values for these symbolic variables. The subsequent section provides a comprehensive exposition of the proof process.

**Theorem** **1.** *The C2BS problem is an NP-hard problem.*

**Proof** **of** **Theorem** **1.** First, we examine a particular case of the C2BS problem, referred to as C2BS*. Furthermore, it is assumed that a single UAV is deployed in this scenario to facilitate coding-based data dissemination for all vehicles throughout the service period, thereby eliminating the need for coordination between multiple UAVs. Based on the above assumptions, the optimal solution to C2BS* is proven to be generated if and only if finding the vectors with the minimum number to achieve full rank simultaneously for all resulting matrices.

The cached packets per vehicle in the set *V* are employed for constructing a collection of matrices represented as *A*. In particular, let $A_{k}={\left[a\left(p_{1}^{k}\right),a\left(p_{2}^{k}\right),\ldots ,a\left(p_{\left|C(v_{k})\right|}^{k}\right)\right]}^{T}$ ($A_{k}\in A$) represet the collection of packets cached by vehicle $v_{k}\left(v_{k}\in V\right)$, as described in Section 4.1 Moreover, it is important to perform Gaussian elimination technology on matrix $A_{k}$ to transform it into its reduced row echelon form, as the vectors in $A_{k}$ may show linear dependence. Then, the transformed matrix, denoted by $A_{k}^{\prime }={\left[a^{\prime }\left(p_{1}^{k}\right),a^{\prime }\left(p_{2}^{k}\right),\ldots ,a^{\prime }\left(p_{|C^{\prime }\left(v_{k}\right)|}^{k}\right)\right]}^{T}$, consists of $|C^{\prime }\left(v_{k}\right)\left||D\right|$-dimension linear dependent vectors, where $|C^{\prime }\left(v_{k}\right)|\leq |C\left(v_{k}\right)|$. Accordingly, the transformation of each matrix in *A* is denoted as $A^{\prime }=\left\{A_{1}^{\prime },A_{2}^{\prime },\ldots ,A_{\left|V\right|}^{\prime }\right\}$.

For matrix $A_{k}^{\prime }\left(A_{k}^{\prime }\in A^{\prime }\right)$, it implies that $|A_{k}^{\prime }|\leq \left|D\right|$, as $A_{k}^{\prime }$ comprises of $|A_{k}^{\prime }|$ coefficient vectors with dimensions of |D|. In particular, the condition $|A_{k}^{\prime }|$ == |D| suggests that matrix $A_{k}^{\prime }$ has full rank and can be converted into an identity matrix by using Gaussian elimination approach. If $|A_{k}^{\prime }\left|\leq |D\right|$, to ensure the acquisition of all data items for vehicle $v_{k}$, it is necessary to append at least $\left|D|-\right|A_{k}^{\prime }|$ coefficient vectors with dimensions of |D| to construct a matrix with full rank. As stated in Section 4.1, a coefficient vector of dimension |D| represents either an encoded or non-encoded packet. The minimum number of packets, denoted as $\left|D|-\right|A_{k}^{\prime }|$, are determined and scheduled to ensure that $v_{k}$ can retrieve all the data items. Then, we define the smallest number of coefficient vectors in $A^{\prime }$ as γ = min $\left(\left\{|A_{1}^{\prime }|,|A_{2}^{\prime }|,\ldots ,|A_{\left|V\right|}^{\prime }\right|\}\right)$. Based on the above analysis, the scheduling of a minimum number of |D|−γ coefficient vectors is necessary to ensure that all matrices in $A^{\prime }$ attain maximum rank. Consequently, the C2BS* problem aims to identify a minimal packet set that can accommodate all vehicles.

Conversely, the optimal solution for the C2BS problem, denoted as $P^{*}=\left\{p_{1}^{*},p_{2}^{*},\ldots ,p_{|P^{*}|}^{*}\right\}$, is assumed to consist of a set of $|P^{*}|$ packets. The set Ω is constructed basen on $P^{*}$, which consists of $|P^{*}|$ coefficient vectors with dimensions of |D| and is denoted as $\Omega =\{a\left(p_{1}^{*}\right),a\left(p_{2}^{*}\right),\ldots ,a\left(p_{|P^{*}|}^{*}\right)\}$. Furthermore, to address the C2BS* problem, the UAV schedules these packets in $P^{*}$ to serve all vehicles. Specifically, we append all coefficient vectors in Ω to each matrix in $A^{\prime }$, resulting in the construction of a collection of matrices denoted by $A^{*}=\left\{A_{1}^{*},A_{2}^{*},\ldots ,A_{\left|V\right|}^{*}\right\}$, where $A_{k}^{*}={\left[a^{\prime }\left(p_{1}^{k}\right),a^{\prime }\left(p_{2}^{k}\right),\ldots ,a^{\prime }\left(p_{|C^{\prime }\left(v_{k}\right)|}^{k}\right),a\left(p_{1}^{*}\right),a\left(p_{2}^{*}\right),\ldots ,a\left(p_{|P^{*}|}^{*}\right)\right]}^{T}$ ($A_{k}^{*}\in A^{*}$). Then, suppose there exists a vehicle $v_{\alpha }$ ($v_{\alpha }\in V$) that does not possess all the data elements. In this case, the matrix $A_{\alpha }^{*}$ ($A_{\alpha }^{*}\in A^{*}$) does not have full rank, indicating the need for additional vectors to construct a matrix with full rank for $A_{\alpha }^{*}$. To accommodate $v_{\alpha }$, it is necessary to schedule more packets. This goes against the previous assumption that $P^{*}$ represents the optimal solution, suggesting that all vehicles can efficiently attain all data items by scheduling these packets in $P^{*}$. It is worth noting that a(p) is a coefficient vector of dimension |D| over GF(2). Consequently, it is equivalent for the C2BS* problem to identifying a minimal collection of coefficient vectors, ensuring that all matrices in $A^{*}$ simultaneously attain full rank.

The above evidence substantiates the NP-hardness of the C2BS* problem, while Theorem 1 establishes a specific case of the C2BS problem as proof.   □

## 5. Proposed Algorithm

### 5.1. Algorithm Design

With the development of optimization algorithms, it is promising to design a dedicated algorithm to solve the complex data dissemination problem in IoV. The genetic algorithm is an optimization algorithm used to simulate the evolution of living things over generations, in which a population of individuals (referred to as candidate solutions ) undergoes evolution towards better individuals. The genetic algorithm has shown competitiveness in solving combinational optimization problems, such as knapsack problem [33], task scheduling [34], and feature selection [35]. Note that the C2BS problem entails the identification and scheduling of a minimal set of packets, wherein each packet can be transformed into a coefficient vector. Hence, the C2BS problem can be considered as a discrete combinatorial optimization problem. In light of this, we propose a GCS algorithm to address the C2BS problem. The following presents the brief procedure of the GCS algorithm.

**Figure 2:**
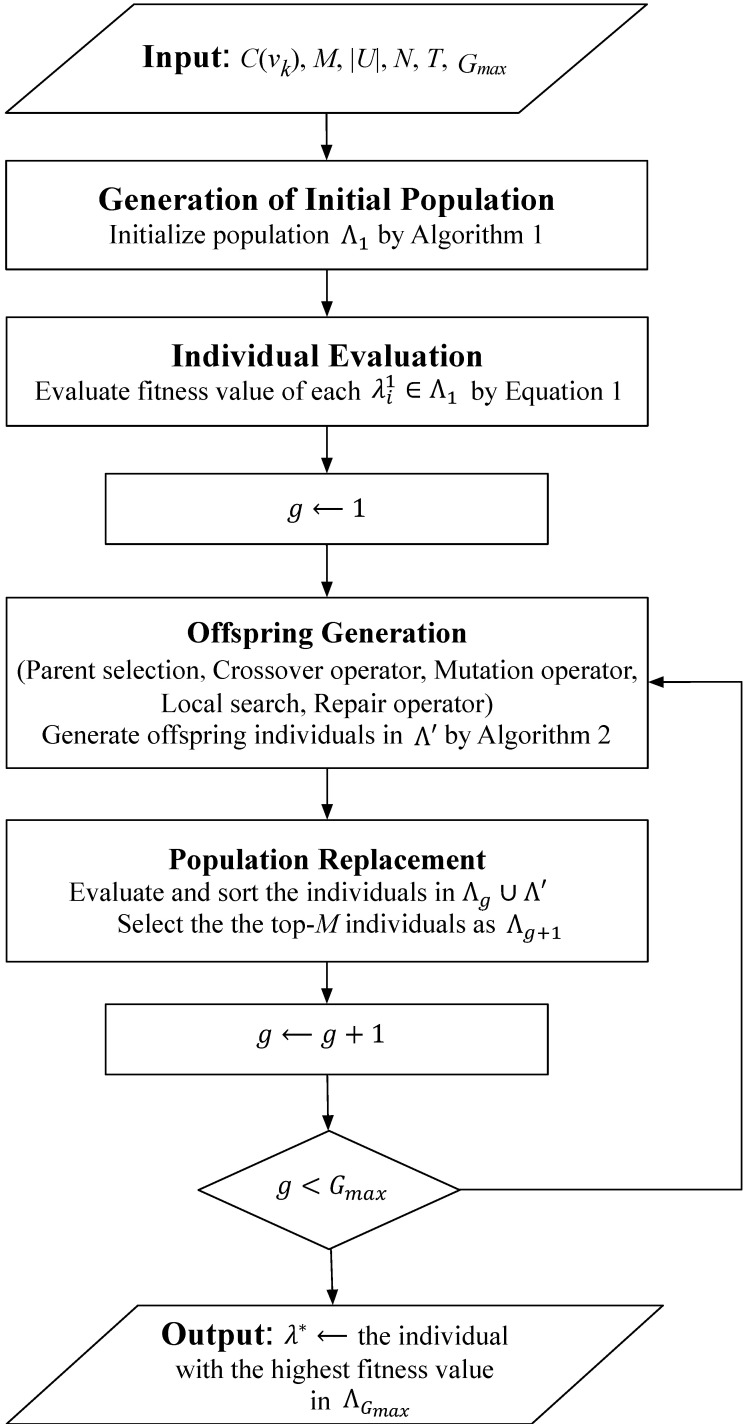
Flow chart of the GCS algorithm.

The GCS algorithm commences with the initialization of a group of individuals, where each individual represents a candidate solution. Furthermore, parent selection is performed based on a dedicated fitness function to determine suitable parents for executing crossover and mutation operators to generate offspring individuals. Moreover, a local search technique is designed to enhance global exploration by leveraging local knowledge, while an effective repair operator is devised to handle infeasible solutions during evolution. Additionally, the offspring population is derived from both parents and the generated offspring solutions through population replacement. The above procedure is iteratively carried out until the predefined termination condition is reached. To better understand the process, we priorly give an overview of the GCS algorithm as a flow chart in Figure 2. The following are the presented steps of the GCS algorithm in detail.

**Figure 3:**
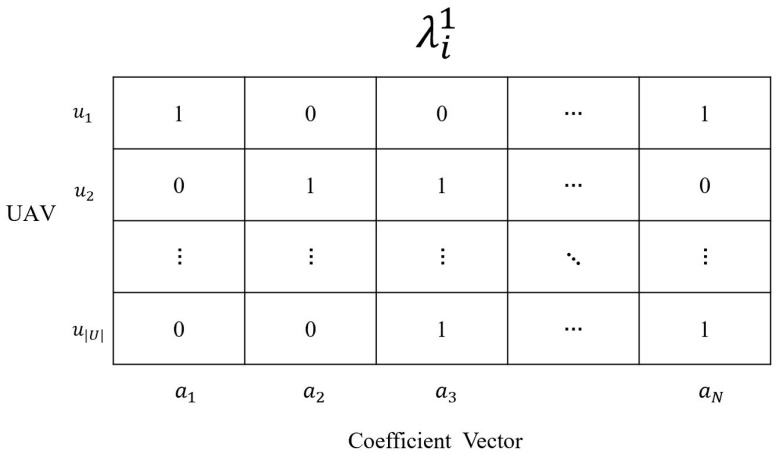
Illustration of the individual $\lambda_{i}^{1}$ in the 1st generation $\Lambda_{1}$.

1. Generation of Initial Population
  A population of individuals is initialized and denoted by $\Lambda_{1}=\left\{\lambda_{1}^{1},\lambda_{2}^{1},\ldots ,\lambda_{M}^{1}\right\}$, where *M* is the size of population. In particular, as shown in Figure 3, an individual $\lambda_{i}^{1}\left(\lambda_{i}^{1}\in \Lambda_{1}\right)$, which represents a candidate solution and is denoted by a binary matrix of size |U|×N, where |U| and *N* denote the overall count of UAVs and the overall count of the randomly generated |D|-dimensional coefficient vectors, respectively. Note that given a coefficient vector $a_{k}=\left[a{\left(k\right)}_{1},a{\left(k\right)}_{2},\ldots ,a{\left(k\right)}_{\left|D\right|}\right]$ (1≤k≤N), $a{\left(k\right)}_{q}$(1≤q≤|D|) is 1 or 0. Then, if $\lambda_{i}^{1}\left[j\right]\left[k\right]$ is set to 1, it means that $a_{k}$ is selected and scheduled by UAV $u_{j}$. Otherwise, $\lambda_{i}^{1}\left[j\right]\left[k\right]=0$. Additionally, the scheduling period allows for a maximum capacity of *T* packets to be scheduled. Given a feasible solution $\lambda_{i}^{1}\left(\lambda_{i}^{1}\in \Lambda^{1}\right)$, each row of $\lambda_{i}^{1}$ should satisfy 0 $\leq \sum_{k=1}^{N}\lambda_{i}^{1}\left[j\right]\left[k\right]\leq T$. The process of population initialization is illustrated within Algorithm 1.
2. Fitness Function
  The coefficient vector a(p) is appended to the coefficient matrix for each vehicle located within the radio coverage range of UAV $u_{j}$ when packet *p* is scheduled by UAV $u_{j}$. Note that the C2BS problem aims to identify and arrange a minimal collection of packets *P* to cater to all vehicles. Then, the following fitness function is designed to evaluate solutions. Specifically, given a solution $\lambda_{i}^{g}\left(\lambda_{i}^{g}\in \Lambda_{g}\right)$, if $\lambda_{i}^{g}\left[j\right]\left[k\right]$ (1≤k≤|U|, 1≤k≤N) is eauql to 1, $a_{k}$ is attached to each vehicle’s coefficient matrix. In a scheduling period, the overall count of packets scheduled by UAV $u_{j}$ is denoted by $b_{j}=\sum_{k=1}^{N}\lambda_{i}^{g}\left[j\right]\left[k\right]$. As shown in Figure 3, for the solution $\lambda_{i}$, the total number of scheduling is represented by $b_{num}=max\left\{b_{1},b_{2},\ldots ,b_{\left|U\right|}\right\}$. Then, according to the solution $\lambda_{i}^{g}$, for each vehicle $v_{k}^{j}\left(v_{k}^{j}\in V_{j}\right)$ in the coverage of the UAV $u_{j}$, let $r(v_{k}^{j})$ and $r^{\prime }\left(v_{k}^{j}\right)$ represent the ranks of original and updated matrices, respectively. After a scheduling period, the average improvement in matrix rank for these vehicles can be calculated by $\sum_{n=1}^{b_{j}}\sum_{k=1}^{|V_{j}|}\left(r^{\prime }\left(v_{k}^{j}\right)-r\left(v_{k}^{j}\right)\right)/\left|V_{j}\right|$. Based on this, we define the following fitness function to evaluate solutions.
  (1) $$f\left(\lambda_{i}^{g}\right)=\sum_{j=1}^{\left|U\right|}(\sum_{n=1}^{b_{j}}\sum_{k=1}^{|V_{j}|}\left(r^{\prime }\left(v_{k}^{j}\right)-r\left(v_{k}^{j}\right)\right)/|V_{j}\left|\right)/b_{num}$$
3. Offspring Generation
  The operators with parent selection, crossover, and mutation are performed to evolve the generation. Specifically, first, the tournament selection scheme [36] is implemented for determining the two parents to allow them to pass their good genes to successive generations. Hence, given the *g*-th generation population $\Lambda_{g}$, the underlying principle of the tournament selection scheme is to prioritize two individuals, denoted as $\lambda_{x}^{g}$ and $\lambda_{y}^{g}$ ($\lambda_{x}^{g},\lambda_{y}^{g}\in \Lambda_{g}$), who exhibit the highest levels of fitness. The parent selection is shown in lines 2∼4 in Algorithm 2. Furthermore, crossover sites are randomly chosen from $\lambda_{x}^{g}$ and $\lambda_{y}^{g}$. Then, a completely new offspring is created by exchanging the genes at these crossover sites. The process of the crossover operator is depicted in lines 5∼10 within Algorithm 2. Additionally, to preserve the variety of the population, the new population undergoes bit mutation with a predetermined probability ρ. The process of mutation operator is illustrated in lines 11∼18 within Algorithm 2.
4. Local Search
  Local search is carried out on each solution in the population to search its neighborhood by steps for a better one. Specifically, given a solution $\lambda_{i}^{g}$ ($\lambda_{i}^{g}\in \Lambda_{g}$), a local search is performed on $\lambda_{i}^{g}$ from the first to the last dimension by conducting a sequential value flipping. Then, through sequential evaluation and comparison of new individuals, only the individual exhibiting the highest fitness value is accepted and saved in the population. The local search is shown in lines 24∼38 within Algorithm 2.
5. Repair Operator
  It is worth noting that an individual may become an infeasible solution in evolution (i.e., crossover operator, mutation operator, and local search). In each scheduling period, the maximum number of packets broadcast by a UAV is *T*. Then, for a solution $\lambda_{i}^{g}$ ($\lambda_{i}^{g}\in \Lambda_{g})$, if $\sum_{k=1}^{N}\lambda_{i}^{g}\left[j\right]\left[k\right]>T$, $\lambda_{i}^{g}$ is an infeasible solution. Hence, we design a repair operator to adjust an infeasible solution. In particular, given a solution $\lambda_{i}^{g}$, we traverse each row of $\lambda_{i}^{g}$, if $\sum_{k=1}^{N}\lambda_{i}^{g}\left[j\right]\left[k\right]>T$, randomly choose $\sum_{k=1}^{N}\lambda_{i}^{g}\left[j\right]\left[k\right]-T$ nonzero elements and set them to 0. Consequently, we have $\sum_{k=1}^{N}\lambda_{i}^{g}\left[j\right]\left[k\right]=T$ by performing the repair operator.
6. Population Replacement
  Population replacement is to update the population and keep the population size steady. To achieve this, new individuals are inserted into the current population. Then, we sort the individuals by their fitness values in descending order. Finally, the top-*M* individuals are selected to form the new population for the next evaluation. The process of population replacement is illustrated in lines 7∼8 within Algorithm 3.

**Algorithm 1** Generation of Initial Population**Input:** *M*, *U*, *N* and *T* **Output:**
$\Lambda_{1}=\left\{\lambda_{1}^{1},\lambda_{2}^{1},\ldots ,\lambda_{M}^{1}\right\}$   1:**for** i=1:M **do**  2:       **for** j=1:|U| **do**  3:            **for** k=1:N **do**  4:                  Assign $\lambda_{i}^{1}\left[j\right]\left[k\right]$ a random value of either 0 or 1.  5:                  **if**   $\sum_{k=1}^{N}\lambda_{i}^{1}\left[j\right]\left[k\right]\geq T$ **then**  6:                           break  7:                  **end if**  8:            **end for**  9:      **end for** 10:**end for**


Algorithm 3 provides a detailed process of the GCS algorithm. The GCS algorithm concludes when it reaches the predefined maximum number of generations $G_{max}$, which is a widely adopted approach in genetic algorithms [37,38]. Consequently, the algorithm outputs the individual $\lambda^{*}$ with the highest fitness value in $G_{max}$-th generation $\Lambda_{G_{max}}$.
**Algorithm 2** Offspring Generation**Input: ***U*, $C(v_{k})$ ($v_{k}\in V$), *T*, and $\Lambda_{g}$ **Output:** the new population $\Lambda^{\prime }=\left\{\lambda_{1}^{\prime },\lambda_{2}^{\prime },\ldots ,\lambda_{M}^{\prime }\right\}$
  1:**for** i=1:M/2  **do***//Parents Selection*  2:     Choose *q* individuals in $\Lambda_{g}$ randomly  3:     Sort them by their fitness value in descending order  4:     Choose the top-2 individuals $\lambda_{x}^{g}$ and $\lambda_{y}^{g}$ as parents*//Crossover Operator*  5:     **for** k=1:N **do**  6:           **for** j=1:|U| **do**  7:               Perform either $\lambda_{2i-1}^{\prime }\left[j\right]\left[k\right]\leftarrow \lambda_{x}^{g}\left[j\right]\left[k\right]$ or $\lambda_{2i-1}^{\prime }\left[j\right]\left[k\right]\leftarrow \lambda_{y}^{g}\left[j\right]\left[k\right]$ randomly  8:               $\lambda_{2i}^{\prime }\left[j\right]\left[k\right]\leftarrow \lambda_{x}^{g}\left[j\right]\left[k\right]+\lambda_{y}^{g}\left[j\right]\left[k\right]-\lambda_{2i-1}^{\prime }\left[j\right]\left[k\right]$  9:           **end for** 10:     **end for***//Mutation Operator* 11:      **for** each offspring individual $\lambda_{l}^{\prime }\in \left\{\lambda_{2i-1}^{\prime },\lambda_{2i}^{\prime }\right\}$ **do** 12:            **for** k=1:N **do** 13:               **for** j=1:|U| **do** 14:                    Generate a number *r* within the range of [0,1] in an arbitrary manner. 15:                    **if** r<ρ **then** 16:                        $\lambda_{l}^{\prime }\left[j\right]\left[k\right]\leftarrow 1-\lambda_{l}^{\prime }\left[j\right]\left[k\right]$ 17:                    **end if** 18:               **end for***//Repair Operator* 19:             **if** $\sum_{k=1}^{N}\lambda_{l}^{\prime }\left[j\right]\left[k\right]>T$ **then** 20:                  perform repair operator on $\lambda_{l}^{\prime }$ 21:             **end if** 22:          **end for** 23:     **end for***//Local Search* 24:     **for** each offspring individual $\lambda_{l}^{\prime }\in \left\{\lambda_{2i-1}^{\prime },\lambda_{2i}^{\prime }\right\}$ **do** 25:         **for** k=1:N **do** 26:            $temp\leftarrow \lambda_{l}^{\prime }$ 27:            **for** j=1:|U| **do** 28:                 $temp\left[j\right]\left[k\right]\leftarrow 1-\lambda_{l}^{\prime }\left[j\right]\left[k\right]$ 29:            **end for** 30:            **if** $\sum_{k=1}^{N}temp\left[j\right]\left[k\right]>T$ **then** 31:                 perform repair operator on temp 32:            **end if** 33:            Evaluate temp by Equation (1) 34:            **if** f(temp) > $f(\lambda_{l}^{\prime })$ **then** 35:               $\lambda_{l}^{\prime }\leftarrow temp$ 36:            **end if** 37:         **end for** 38:      **end for** 39:**end for**


**Algorithm 3:** The GCS algorithm**Input: ***M*, *U*, $C(v_{k})$ ($v_{k}\in V$), *N*, *T*, $G_{max}$ **Output:**
$\lambda^{*}$ with the highest fitness value in $G_{max}$-th generation $\Lambda_{G_{max}}$ *   //Generation of initial population*
  1:g=1  2:$\Lambda_{1}\leftarrow$ Generate a initial population by Algorithm 1  3:Evaluate fitness value $f(\lambda_{i}^{1})$ of each $\lambda_{i}^{1}\in \Lambda_{1}$ by Equation (1)  4:**while **$g<G_{max}$** do***//Offspring Generation*  5:      $\Lambda^{\prime }\leftarrow$ Generate a new population by Algorithm 2*//Population replacement*  6:      Evaluate fitness value of each individuals in $\Lambda_{g}\cup \Lambda^{\prime }$ by Equation (1)  7:      Sort the individuals in $\Lambda_{g}\cup \Lambda^{\prime }$ by their fitness value in descending order  8:      $\Lambda_{g+1}\leftarrow$ Top-*M* individuals in $\Lambda_{g}\cup \Lambda^{\prime }$  9:      g←g+1 10:**end while** 11:Output the individual with the highest fitness value in $\Lambda_{G_{max}}$


### 5.2. Time Complexity Analysis

The time complexity of the GCS algorithm is analyzed according to its procedural steps. For the population initialization, since a solution $\lambda_{i}^{1}\left(\lambda_{i}^{1}\in \Lambda_{1}\right)$ is a |U|×N binary matrix, it traverses |U|×N bits to construct. The time overhead for initializing the population is O(|U|×N×M), with *M* representing the size of the population. In offspring generation, for the parent selection operator, we randomly choose *k* individuals and sort them by their fitness values in descending order. According to Algorithm 2, the operation is required for matrices of |V| vehicles, with each matrix having a maximum size of |D|×|D|. The crossover operator requires traversing two offspring individuals with a time overhead of 2×|U|×N. For the mutation operator, it is also required to traverse two offspring individuals. For the repair operator, at worst *N* times repair operator is required to be implemented for each offspring individual. Based on this, the time overhead for generating offspring is $O(k\times |V|\times |D{|}^{2}+2\times N+2\times N+2\times |U|\times N^{2})$, which can be further simplified to $O(|V|\times |D{|}^{2}+|U|\times N^{2})$. The local search requires operating on each bit in the two offspring individuals, applying the repair operator to them, and evaluating their fitness. Consequently, the time overhead of the local search can be expressed as $O(2\times N+2\times |U|\times N^{2}+2\times N\times |V|\times |D{|}^{2})$, which can be further simplified to $O(N\times |V|\times |D{|}^{2}+|U|\times N^{2})$. The loop termination condition is determined by $G_{max}$.

Based on the analysis provided above, it can be inferred that the GCS algorithm exhibits a time complexity of $O(|U|\times N\times M)+O(G_{max}\times \left(|V\right|\times {\left|D\right|}^{2}+\left|U\right|\times N^{2}))+O(G_{max}{\times (N\times |V|\times |D|}^{2}+\left|U\right|\times N^{2}))$, that is $O(|U|\times N\times M+G_{max}{\times (N\times |V|\times |D|}^{2}+\left|U\right|\times N^{2}))$. The parameters |U|, *N*, *M*, and $G_{max}$ are treated as constants in the GCS algorithm, with their values independent of the system’s scale. Then, the time complexity has been decreased to $O(|V|\times |D{|}^{2})$. Additionally, considering |D| as a constant or small scale in specific scenarios, such as post-disaster rescue, is justifiable due to its representation of the database size. The time complexity of the GCS algorithm exhibits a robust linear correlation with the number of vehicles in the service scenario, thereby rendering it practically applicable for addressing the C2BS problem in terms of time complexity.

## 6. Performance Evaluation

### 6.1. Simulation Setup

**Figure 4:**
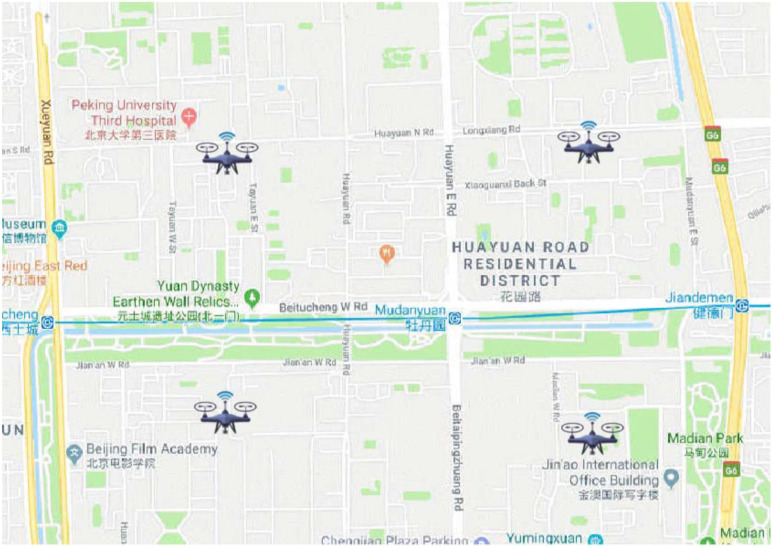
Simulation scenario.

We conduct a simulation model utilizing the system architecture outlined in Section 3. In particular, we extract taxi trajectories from a 3 km × 3 km region located in Haidian District, Beijing, China, during the time frame of 23:50 to 23:55 on 13 November 2015 to accurately mimic real-world scenarios, as shown in Figure 4. The extracted trajectories consist of a total of 277 vehicles with an average residence time of 213.6 s and a standard deviation in residence time of 10 s. Additionally, the average vehicle number per second is 198, with a standard deviation of 13.3 in terms of the number of vehicles. Moreover, four UAVs are deployed in simulation for data dissemination, while the algorithm is implemented using MATLAB R2021a.

To facilitate performance comparison, we implement three competitive algorithms. The first algorithm, MRF [39], employs a non-coded scheduling strategy, aiming to prioritize the data item with the highest count in the pending request queue. The second one, MRF_C [40], utilizes an encoded packet scheduling approach to broadcast pending requests with the maximum number. The last one, Round-Robin [41], adopts a sequential broadcasting scheme to transmit all data items in rotation. The default settings for the GCS algorithm comprise a population size of 100, assigning a total number of solution vectors *N* as 30, limiting the maximum number of iterations $G_{max}$ to 100, fixing the mutation probability ρ at 0.01, and restricting the maximum scheduling period *T* to 5. Additionally, the transmission rate of the UAV is configured to be 6 Mb/s, while the communication radius is set at 400 m. The database size is defined as 60, with each data item having a size of 18 Mb. Upon entering the simulation area, vehicles may possess cached data items that adhere to a Zipf distribution with a skewness parameter of 0.8 and a cache ratio set at 0.6 (referring to the ratio between total cached data items and those in the database). Unless otherwise specified, all simulations are conducted under these default settings.

The performance of algorithms is quantitatively evaluated by establishing metrics as follows.

- Average Service Delay (ASD): The delay in service for vehicle $v_{k}$ ($v_{k}\in V$) (denoted by $t_{k}$) is indicated as the duration between entering the simulated area and obtaining all data items in the database. The ASD is computed as the sum of service delays for all vehicles divided by the overall count of vehicles, as follows:
  (2) $$ASD=(\sum_{k=1}^{\left|V\right|}t_{k})/|V|$$
- Average Service Productivity (ASP): Let $r_{k}$ and $r_{k}^{\prime }$ denote the ranks of the original and new coefficient matrices, respectively, for vehicle $v_{k}\in V$, while $n_{k}$ represents the number of scheduling packets during its service. The ASP is calculated as the sum of the improved rank for each vehicle per scheduling packet divided by the overall count of vehicles, as follows:
  (3) $$ASP=(\sum_{k=1}^{\left|V\right|}\frac{r_{k}-r_{k}^{\prime }}{n_{k}})/|V|$$
- Average Service Ratio (ASR): After the simulation, let *s* denote the total count of vehicles that successfully retrieve all data items. Then, the SR is calculated as follows:
  (4) SR=s/|V|

### 6.2. Performance Analysis

#### 6.2.1. Effect of Cache Ratio

**Figure 5:**
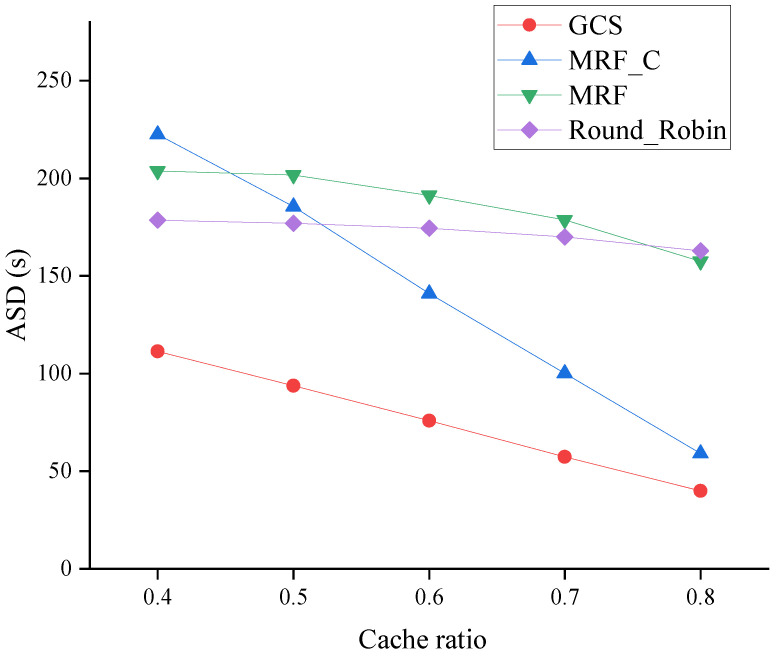
ASD across varying cache ratios.

**Figure 6:**
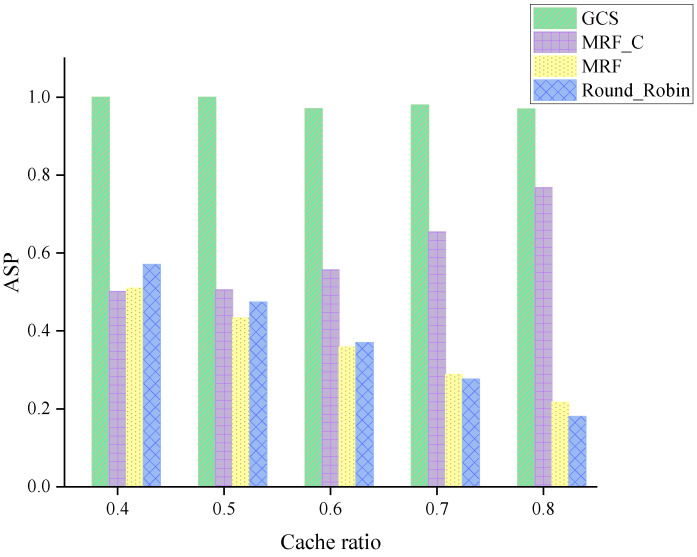
ASP across varying cache ratios.

**Figure 7:**
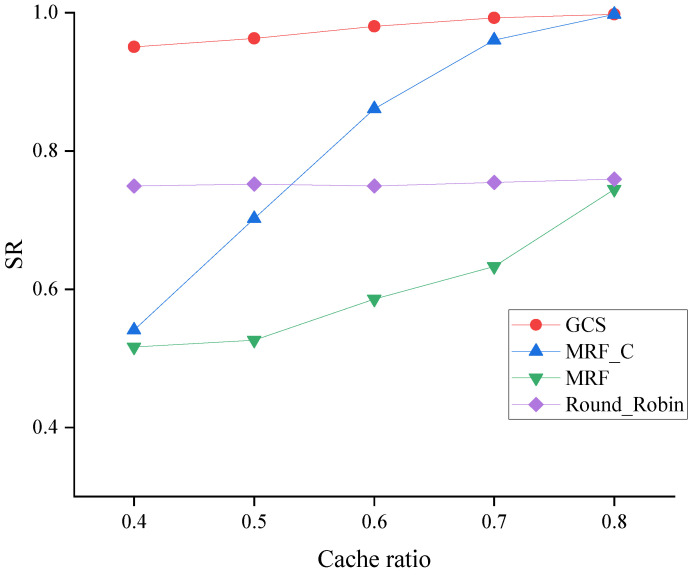
SR across varying cache ratios.

The performance evaluation of algorithms across varying cache ratios is illustrated in Figure 5, Figure 6 and Figure 7. Specifically, the ASD of four algorithms across varying cache ratios is depicted in Figure 5. According to Figure 5, an increase in the cache ratio results in a decrease in the ASD of all algorithms. The higher cache ratio results in more packets being cached by vehicles, reducing the overall system workload. The GCS exhibits superior performance across all ranges, thereby demonstrating the efficacy of its approach by leveraging the advantages of coding and caching in multi-UAV data dissemination scenarios.

Figure 6 compares the ASP of four algorithms for various cache ratios. A higher ASP indicates on average more vehicles are served through per scheduling. As shown in Figure 6, the GCS consistently endeavors to uphold the upper limit of the ASP across various scenarios. Furthermore, it has been noted that the MRF and the Round-Robin underperform in terms of the ASP. As the cache ratio increases, vehicles cache a larger number of packets, resulting in a reduction in the number of vehicles served with non-encoded packets. Additionally, with the cache ratio gradually increasing, the difference between GCS and MRF_C is becoming less significant. The reason lies in the benefits of network coding. With an encoded packet, more vehicles can be served. This result confirms findings in [40].

The SR of four algorithms across varying cache ratios is depicted in Figure 7. When the system works with a higher cached ratio, vehicles cache more packets, and all the algorithms should have better performance on SR. As illustrated in Figure 7, when the cache ratio is increased, the SR of all algorithms is raised. Moreover, the comparison results indicate that MRF_C outperforms MRF, thereby demonstrating the potential of MRF_C to enhance bandwidth efficiency in UAVs through network coding. In addition, comparing the GCS with the MRF_C, the result shows the superiority of the GCS by exploiting the benefit of coding and caching.

#### 6.2.2. Effect of Database Sizes

**Figure 8:**
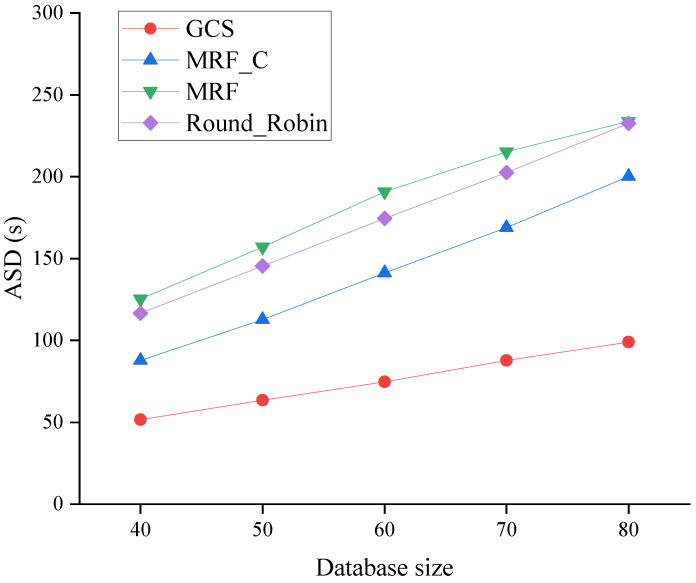
ASD across varying database sizes.

**Figure 9:**
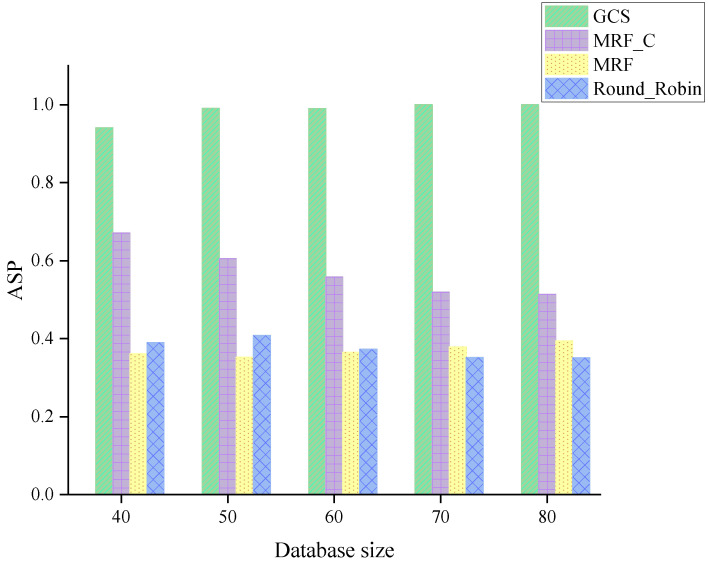
ASP across varying database sizes.

**Figure 10:**
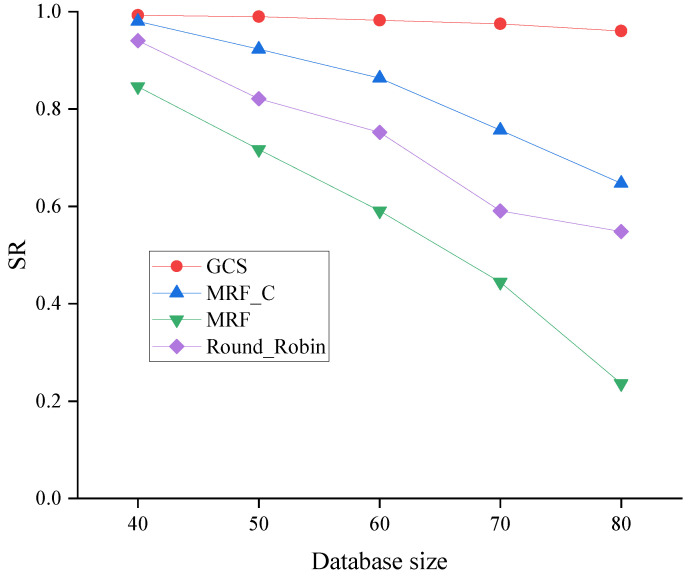
SR across varying database sizes.

Figure 8, Figure 9 and Figure 10 show the performance of algorithms across varying database sizes. Specifically, the ASD of four algorithms is compared with different database sizes in Figure 8. As depicted in Figure 8, the ASD of all algorithms exhibits an increase when the database size expands. The expansion of the database size leads to an increase in the overall system workload. Nevertheless, the GCS consistently exhibits the lowest ASD compared to other algorithms across all scenarios.

The ASP of four algorithms across varying database sizes is illustrated in Figure 9. An algorithm with higher ASP states the better performance on improving the bandwidth efficiency. As illustrated in Figure 9, GCS consistently outperforms other algorithms across all scenarios. The MRF_C outperforms the MRF and the Round_Robin by leveraging coding-based scheduling decisions.

Figure 10 compares the SR of four algorithms across varying database sizes from 40 to 80. According to Figure 10, the decrease in the SR of all algorithms is observed with an increase in the database size, owing to the necessity of scheduling more packets to serve all vehicles when dealing with a large-scale database. However, the GCS consistently demonstrates superior performance in terms of the SR across all ranges, which further demonstrates the effectiveness of the GCS by making full use of coding and caching.

#### 6.2.3. Effect of Data Sizes

**Figure 11:**
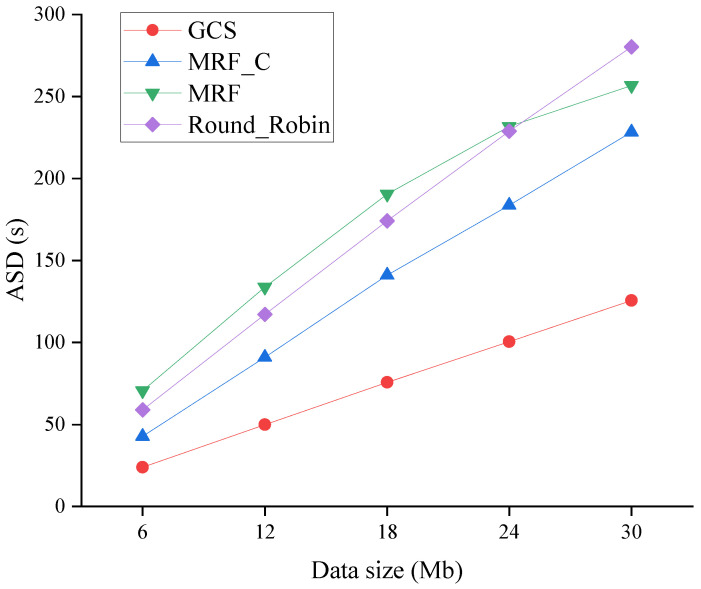
ASD across varying data sizes.

**Figure 12:**
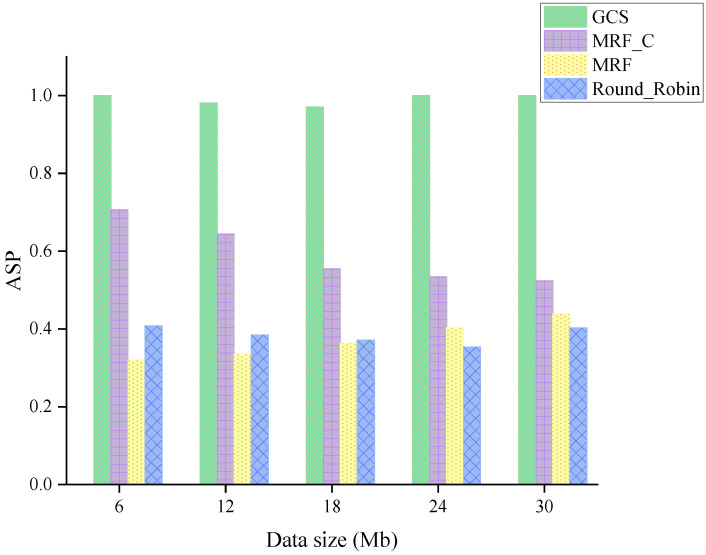
ASP across varying data sizes.

**Figure 13:**
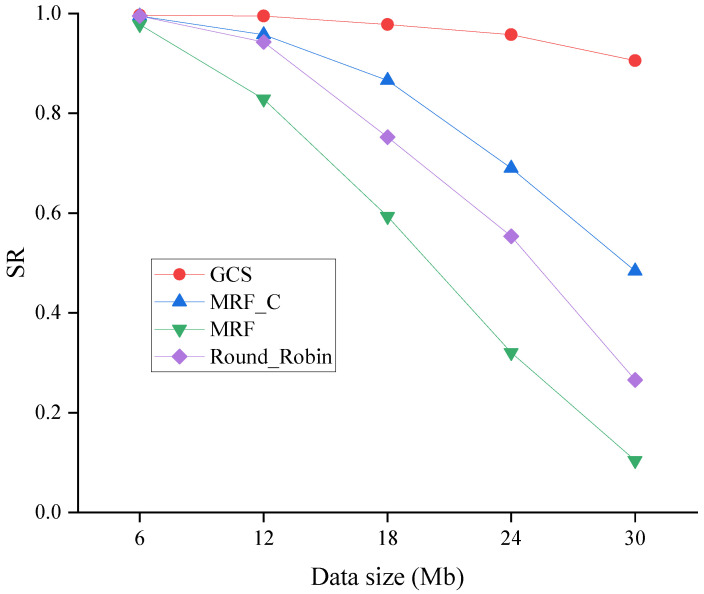
SR across varying data sizes.

Figure 11, Figure 12 and Figure 13 evaluate the performance of four algorithms across varying data sizes. Specifically, Figure 11 compares the ASD of four algorithms across varying data sizes. Due to the fixed transmission rate, the increase in data size leads to a proportional escalation of transmission delay, thereby imposing a higher burden on the system workload. As shown in Figure 11, when the data size is increased, the ASD gets higher. However, the GCS still exhibits the lowest ASD.

Figure 12 exhibits the ASP of four algorithms across varying data sizes. As shown in Figure 12, the heavy system workload prevents four algorithms from completing service before vehicles leave the scenario. However, the GCS demonstrates superior performance in terms of optimizing bandwidth efficiency compared to other algorithms. Moreover, when the data size continues to increase, the disparity between the GCS and the MRF_C becomes more pronounced, thereby highlighting the superiority of the GCS.

Figure 13 shows the SR of four algorithms across varying data sizes. As shown in Figure 13, the SR of four algorithms is decreased when the data size becomes bigger. However, the GCS consistently maintains the highest success rate across all scenarios, thereby further substantiating its efficacy and scalability.

## 7. Conclusions

This work investigated a data dissemination framework integrated SDN in multi-UAV-enabled IoV. Based on this framework, the C2BS problem was formulated to maximize UAVs’ bandwidth efficiency for enhancing overall system performance. Then, we proved that the C2BS problem is an NP-hard problem and proposed a scheduling algorithm based on the genetic algorithm to address the C2BS problem. Additionally, the time complexity of the GCS algorithm was analyzed in order to demonstrate its practicality. Finally, the performance of the GCS algorithm was evaluated in comparison with alternative algorithms. The simulation findings show the effective performance of the proposed scheme in enhancing UAVs’ bandwidth efficiency and optimizing the performance of the system.

As future work, our research can be extended by considering collaboration between UAVs and existing infrastructures (e.g., BSs and RSUs) to enhance scalability in UAV-enabled IoV scenarios. Moreover, we will investigate efficient algorithms by considering the features and constraints of UAVs to enhance the performance of multi-UAV enable IoV.

## Figures and Tables

**Table 1:** The results of vehicle trajectory prediction.

	Time Slot	$t_{0}$	$t_{1}$	$t_{2}$
UAV				
UAV_1_		$v_{1}$, $v_{2}$, $v_{3}$, $v_{4}$	$v_{1}$, $v_{2}$	
UAV_2_		$v_{5}$, $v_{6}$	$v_{3}$, $v_{4}$, $v_{5}$, $v_{6}$	$v_{1}$, $v_{2}$, $v_{3}$, $v_{4}$, $v_{5}$, $v_{6}$

**Table 2:** The different scheduling strategies.

	$t_{0}$	$t_{1}$	$t_{2}$	Average Delay
	UAV_1_: $d_{1}$	UAV_1_: $d_{3}$	UAV_1_: ∅	2.8 time slots
IS	UAV_2_: $d_{1}$	UAV_2_: $d_{2}$	UAV_2_: $d_{4}$	
	UAV_1_: $d_{1}⊕d_{4}$	UAV_1_: $d_{2}⊕d_{3}$	UAV_1_: ∅	2.3 time slots
CIS	UAV_2_: $d_{1}⊕d_{2}$	UAV_2_: $d_{3}⊕d_{4}$	UAV_2_: $d_{1}⊕d_{2}$	
	UAV_1_: $d_{1}⊕d_{4}$	UAV_1_: $d_{3}$	UAV_1_: ∅	2 time slots
CCS	UAV_2_: $d_{3}⊕d_{4}$	UAV_2_: $d_{1}⊕d_{2}$	UAV_2_: ∅	

**Table 3:** Overview of symbols.

Symbols	Descriptions
*V*	The set of vehicles
*D*	The set of data items in the database
*U*	The set of UAVs
*p*	An encoded or non-encoded packet
$C(v_{k})$	The set of packets cached by vehicle $v_{k}$
$A_{k}$	The coefficient matrix of vehicle $v_{k}$ constructed by $C(v_{k})$
*T*	The maximum scheduling period
$V_{j}$	The set of vehicles in the radio coverage range of UAV $u_{j}\left(u_{j}\in U\right)$
$\Lambda_{g}$	The *g*-th generation population
*M*	The total count of individuals in the population
$\lambda_{i}^{g}$	An individual in the *g*-th generation population $\lambda_{i}^{g}\in \Lambda_{g}$
*N*	The total count of *D*-dimensional coefficient vectors
$r(v_{k})$	The rank of matrix $A_{k}$ for $v_{k}$
$f(\lambda_{i}^{g})$	The fitness function for evaluating individual $\lambda_{i}^{g}$ ($\lambda_{i}^{g}\in \Lambda_{g}$)
$G_{max}$	The maximum count of generations

## Data Availability

The data presented in this study are available on request from the first author.
